# The feasibility of a Bayesian network model to assess the probability of simultaneous symptoms in patients with advanced cancer

**DOI:** 10.1038/s41598-022-26342-4

**Published:** 2022-12-24

**Authors:** Lotte van der Stap, Myrthe F. van Haaften, Esther F. van Marrewijk, Albert H. de Heij, Paula L. Jansen, Janine M. N. Burgers, Melle S. Sieswerda, Renske K. Los, Anna K. L. Reyners, Yvette M. van der Linden

**Affiliations:** 1 Center of Expertise in Palliative Care, Leiden University Medical Center, Albinusdreef 2, PO box 9600, 2300 RC Leiden, The Netherlands; 2 Department of Radiology and Nuclear Medicine, Erasmus Medical Center, University Medical Center Rotterdam, Rotterdam, The Netherlands; 3 Department of BioMechanical Engineering, Faculty of Mechanical, Maritime and Materials Engineering, Delft University of Technology, Delft, The Netherlands; 4 Center of Expertise for Palliative Care, University of Groningen, University Medical Center Groningen, Groningen, The Netherlands; 5 Department of Medical Oncology, University of Groningen, University Medical Center Groningen, Groningen, The Netherlands; 6 Faculty of Medicine, Leiden University Medical Center, Leiden, The Netherlands; 7 Faculty of Medicine, Erasmus Medical Center, University Medical Center Rotterdam, Rotterdam, The Netherlands; 8 Department of Research and Development, Netherlands Comprehensive Cancer Organization, Utrecht, The Netherlands; 9 Department of Radiation Oncology (MAASTRO), GROW School for Oncology and Developmental Biology, Maastricht University Medical Center, Maastricht, The Netherlands; 10 Department of Medical Informatics, Erasmus Medical Center, University Medical Center Rotterdam, Rotterdam, The Netherlands; 11 Netherlands Comprehensive Cancer Organisation, Utrecht, The Netherlands

**Keywords:** Cancer, Oncology, Signs and symptoms

## Abstract

Although patients with advanced cancer often experience multiple symptoms simultaneously, clinicians usually focus on symptoms that are volunteered by patients during regular history-taking. We aimed to evaluate the feasibility of a Bayesian network (BN) model to predict the presence of simultaneous symptoms, based on the presence of other symptoms. Our goal is to help clinicians prioritize which symptoms to assess. Patient-reported severity of 11 symptoms (scale 0–10) was measured using an adapted Edmonton Symptom Assessment Scale (ESAS) in a national cross-sectional survey among advanced cancer patients. Scores were dichotomized (< 4 and ≥ 4). Using fourfold cross validation, the prediction error of 9 BN algorithms was estimated (Akaike information criterion (AIC). The model with the highest AIC was evaluated. Model predictive performance was assessed per symptom; an area under curve (AUC) of ≥ 0.65 was considered satisfactory. Model calibration compared predicted and observed probabilities; > 10% difference was considered inaccurate. Symptom scores of 532 patients were collected. A symptom score ≥ 4 was most prevalent for fatigue (64.7%). AUCs varied between 0.60 and 0.78, with satisfactory AUCs for 8/11 symptoms. Calibration was accurate for 101/110 predicted conditional probabilities. Whether a patient experienced fatigue was directly associated with experiencing 7 other symptoms. For example, in the absence or presence of fatigue, the model predicted a 8.6% and 33.1% probability of experiencing anxiety, respectively. It is feasible to use BN development for prioritizing symptom assessment. Fatigue seems most eligble to serve as a starting symptom for predicting the probability of experiencing simultaneous symptoms.

## Introduction

Most patients with advanced cancer experience multiple symptoms simultaneously^1^. However, in daily practice the focus of symptom management is often on one or few main symptoms, that is, the symptoms that are spontaneously volunteered by the patient during regular history-taking^2,3^. This causes other simultaneous symptoms to remain unrelieved, which negatively impacts a patient’s functioning and quality of life^4^. Several solutions have been suggested for assessing symptom burden more comprehensively. Most notably, symptom assessment scales have shown to improve the assessment of total symptom burden^5,6^ but it is difficult to widely implement such scales in both specialist and non-specialist palliative care settings^7^. In addition, efforts were made to identify which symptoms frequently occur simultaneously in patients with advanced cancer, so called symptom clusters^8^. Symptom cluster research has advanced the way simultaneous symptoms are approached theoretically, for example by fostering research about common etiologies of clusters^9^. However, symptom clusters are inconsistent across studies^8^ and, perhaps as a result, cluster research has not yet convincingly impacted symptom assessment in daily practice^9^.

It could help clinicians prioritize which symptoms to assess if they are provided with the probability that their patient experiences specific simultaneous symptoms, based on volunteered main symptoms. For this purpose, a Bayesian network (BN) may be developed. A BN is a probabilistic graphical model used to visualize associations between large numbers of variables. In addition, in case of dichotomized variables, a BN can provide the conditional probability that a variable is present or absent, based on the presence or absence of other variables in the network. BNs have been widely used in medicine to predict outcomes such as diagnosis, functional outcome, quality of life and survival, based on patient and disease characteristics^10–15^. The advantage compared to other probabilistic modelling methods is that they do not need dedicated input and output variables and that they can be constructed in case of insufficient available evidence on associations between variables^15^. BNs are also easy to understand: The graphical structure makes associations between variables directly interpretable and the provided conditional probabilities align with clinical reasoning^15,16^. The aim of this study is to evaluate the statistical feasibility of a BN model for predicting the probability of a simultaneously occurring symptom, based on a patient’s other symptoms.

## Methods

### Context

This study is part of the Multidimensional Strategy for Palliative Care (MuSt-PC) project (2017–2021; NCT03665168). The project aims to improve multidimensional symptom management in palliative care by studying the prevalence of multidimensional symptoms in a national cross-sectional study^17^, evaluating the acceptability of a clinical decision support system (CDSS) according to various stakeholders^18^, assessing barriers and facilitators for multidimensional symptom management^19^, developing symptom management recommendations for simultaneously occurring symptoms and constructing a CDSS to support generalist clinicians.

### Participants and study design

Data on symptom presence and severity were collected during four weeks in September and November 2018 in a nation-wide cross-sectional survey among patients with palliative care needs, regardless of their underlying illness. Physicians and nurses working in different care settings were asked to participate in data collection (general practices, nursing homes, hospices and outpatient departments and clinical wards of academic and community hospitals). Clinicians were asked to identify patients with palliative care needs, using a negative answer to the one-year surprise question as the sole inclusion criterion (answer “No” to the question “Would I be surprised if this patient died within the next twelve months?”)^20^. Patients unable or unwilling to self-assess their symptoms were excluded. In this study we performed a secondary analysis of collected data of patients with advanced cancer.

### Measurements

In case of eligibility, the attending clinician asked their patient to complete a questionnaire to report their symptoms at the time of consultation. The questionnaire included the Utrecht Symptom Diary (USD), a validated Dutch translation and adaptation of the Edmonton Symptom Assessment System (ESAS)^21^. The USD measures presence and severity of 11 symptoms on a 0-to-10-numeric rating scale (NRS) (0 = ‘no complaint’; 10 = ‘worst complaint possible’): Pain, sleeping problems, dry mouth, dysphagia, lack of appetite, constipation, nausea, shortness of breath, fatigue, anxiety and depressed mood. Additionally, the questionnaire contained questions on demographic, treatment and disease characteristics, including a Patient Reported Performance Status (PRFS; scale 1–4). A PRFS 1 indicated ‘not my normal self, but able to be up and about with fairly normal activities’ and a PRFS 4 indicated ‘pretty much bedridden, rarely out of bed’ (see Table 1)^22^. Questionnaires were available via a secured website or on paper.

### Statistical analysis

Frequencies of demographic, disease and treatment characteristic were analyzed using descriptive statistics. In case of missing values, symptom scores (NRS 0–10) were estimated using k-nearest neighbor imputation (k = 5)^23^. Scores were dichotomized into clinically relevant (≥ 4) and not clinically relevant (< 4), since an ESAS score of ≥ 4 is generally considered as the cut-off point for symptoms that require additional assessment^21,24^. For each of the USD-listed symptoms, the frequency of clinically relevant symptom scores was calculated.

#### Bayesian network development

A Bayesian network (BN) was developed to assess whether the presence or absence of a clinically relevant USD-listed symptom could be predicted based on the presence or absence of the other 10 USD-listed symptoms. The dichotomized symptom scores of the 11 USD-listed symptoms served as the network’s variables.

**Figure 1:**
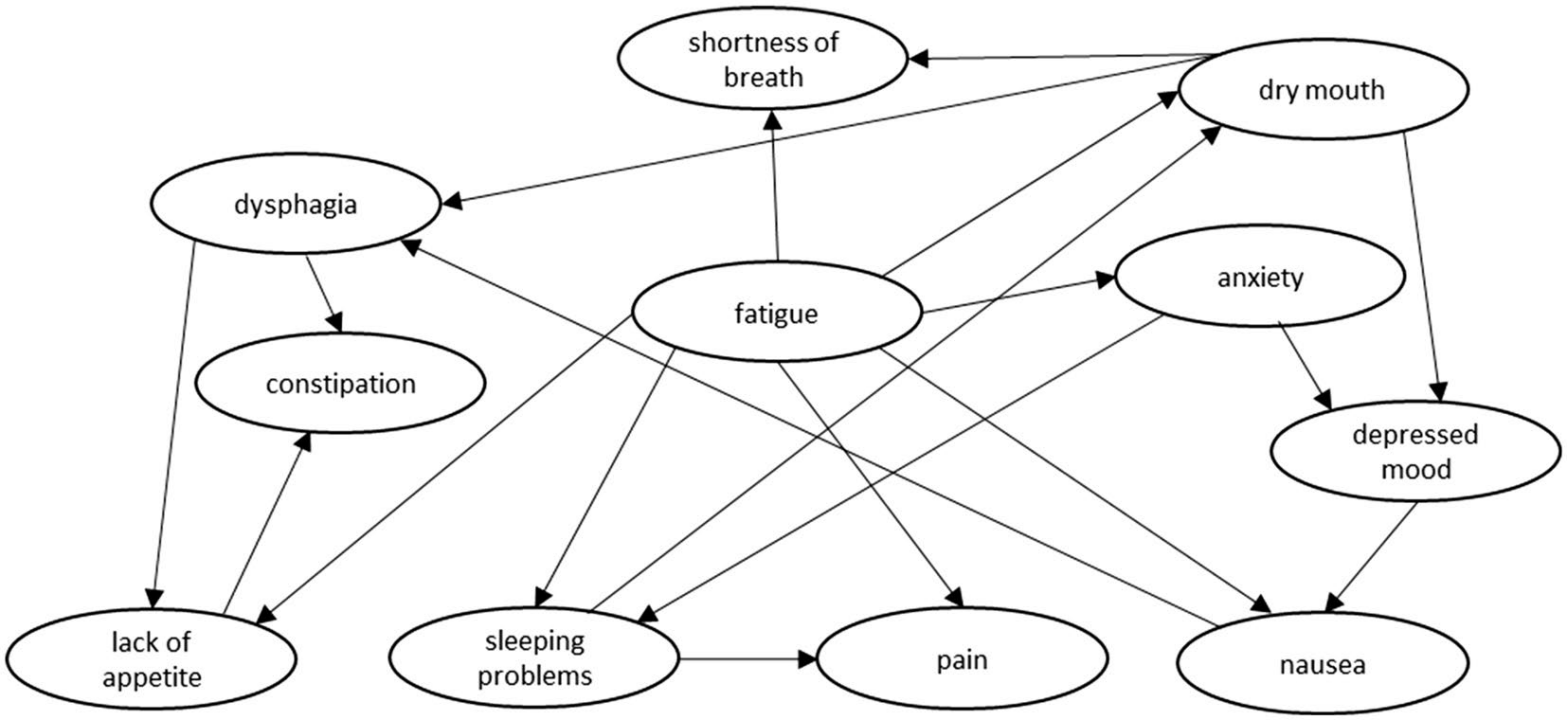
Directed acyclic graph of the Baysian network; as constructed during structure learning by the algorithm with the lowest prediction error estimate (Tabu search algorithm). Edges (arrows) between nodes indicate that the model found a direct association between those variables. An edge points from a parent node towards a child node, indicating that the BN structure found that the child node is conditionally dependent on the parent node.

BN development consists of two stages: Structure learning and parameter learning. During structure learning, a graphical structure is constructed: The directed acyclic graph (DAG) (Fig. 1). In the DAG, edges point from parent nodes towards child nodes, indicating that the model found a direct association, or conditional dependency, between those variables. The DAG presented in this study is based on the total dataset as a way of presenting the mean DAG for our data. All other analyses were conducted per fold within a fourfold cross validation set-up.

Structure learning was done using 9 automated BN algorithms (constraint-based, score-based and hybrid learning). For each of the four resulting DAGs, the Akaike information criterion (AIC) score was calculated. The AIC indicates how accurate a model will be able to predict future data, but only in comparison to other algorithms. The highest AIC score denoted the lowest prediction error estimate^25^.

The algorithm with the highest mean AIC across the four folds was then used for parameter learning. During parameter learning, a conditional probability table (CPT) is calculated for each dichotomized symptom in the model. In theory, conditional probabilities of the presence or absence of each symptom can be calculated, based on the presence or absence of all other variables that the symptom is directly or indirectly connected with in the DAG. In this study, we labelled symptoms that the model identified as parent nodes as ‘main symptoms’ and labelled child nodes as ‘simultaneous symptoms’. We only present the predicted probabilities of the presence of child nodes based on the presence or absence of parent nodes. That way, we limited the number of analyses to illustrate the use of a BN model in a simplified manner.

#### Predictive performance and calibration of the Bayesian network

For the algorithm with the highest AIC score, a mean AUC score over the four folds was calculated per simultaneous symptom and accepted as the model’s overall AUC-ROC. The AUC-ROC indicates how well the BN model can determine the outcome for an individual patient (simultaneous symptom present or absent), based on the presence or absence of the other 10 USD-listed symptoms. In general, an AUC-ROC of 0.5 implies a lack of predictive performance and an AUC-ROC of > 0.9 implies outstanding predictive performance^26^. An AUC-ROC of ≥ 0.65 was considered satisfactory. We also aimed to determine how accurate the BN was in predicting the conditional probabilites of a patient experiencing each simultaneous symptom, based on the presence or absence of the other 10 USD-listed symptoms. For this purpose, a calibration plot was constructed for each symptom using the model's predictions for all four test sets of the cross validation. Patients in the dataset were grouped into deciles, based on their similar predicted conditional probabilities of experiencing the symptom by the BN model. The mean conditional probability predicted by the model was plotted against the mean observed frequency in the dataset per decile. A > 10% difference between predicted and observed conditional probabilities was considered an inaccurate calibration. R 4.1.0 and bn learn R package 4.7 were used to conduct analyses.

#### Development of a symptom prediction system

We aimed to indicate the potential of using a BN model for the development of a symptom prediction system. Based on the BN, we propose a preliminary symptom prediction flow-chart that uses main symptoms that can directly predict the presence or absence of simultaneous symptoms as system input. In addition, the system’s input should preferably consist of those symptoms that are most frequently volunteered by patients with advanced cancer after open-ended questioning during regular history-taking. The only available study on this subject by Homsi et al. identified that pain and fatigue are the most frequently volunteered symptoms, by 50.5% and 25.5% of patients with advanced cancer, respectively. Pain and fatigue were followed by anorexia (13.5%) and constipation (5.5%)^5^.

#### Ethical considerations

The Medical Ethics Review Board of the University Medical Center Groningen approved the research protocol (NCT number 03665168) and waived informed consent (12 June 2018). Data in this study were anonymously obtained and recorded. The opt-in method was used with an anonymous consent statement for study participation and publication of data, to ensure no personal information was reported. The study was conducted in full compliance with the codes of ethical conduct from the 1964 Declaration of Helsinki.

## Results

### Baseline characteristics

The survey was returned by 532 patients with advanced cancer. Twenty-six questionnaires had 1 to 3 missing scores, resulting in 36 missing symptom scores and 5816 complete symptom scores. An overview of baseline characteristics is shown in Table 1. The most frequently reported primary cancer site was lung (19.0%), colon (15.2%), and breast (13.0%), and 70% had metastatic cancer. In the 3 months prior to data collection, 49.4% received systemic therapy and 24.8% radiotherapy. A patient-reported functional status (PRFS)^22^ of 1 was reported by 33.5%; a PRFS of 3 or 4 was reported by 35.8%. At the time of the survey, 51% of patients resided at home.

**Table 1:** Baseline characteristics and symptom burden of participants with advanced cancer, who filled out the cross-sectional symptom assessment survey.

Characteristics	Participants n = 532
	n (%)
**Age category**	
30–45 years	15 (2.8)
46–67 years	208 (39.1)
68–80 years	222 (41.7)
≥ 81 years	84 (15.8)
Unknown	3 (0.6)
**Gender**	
Male	277 (52.1)
Female	252 (47.4)
Unknown	3 (0.6)
**Primary cancer site**^**a**^	
Breast	69 (13.0)
Melanoma	16 (3.0)
Lymphatic system/ leukemia	17 (3.2)
Bladder/urinary tract	23 (4.3)
Colon	81 (15.2)
Lung	101 (19.0)
Prostate	56 (10.5)
Kidney	14 (2.6)
Endometrium	6 (1.1)
Ovaries	16 (3.0)
Pancreas	34 (6.4)
Head/neck	23 (4.3)
Esophagus	15 (2.8)
Liver	10 (1.9)
Brain	13 (2.4)
Neuroendocrine tumours	10 (1.9)
Other^b^	71 (13.3)
**Metastases**	
Yes	373 (70.1)
**Disease modifying treatment during last 3 months**	
Systemic therapy^c^	263 (49.4)
Radiotherapy	132 (24.8)
Surgery	44 (8.3)
No	192 (36.0)
**Patient-reported functional status**^**d**^	
0	61 (11.5)
1	178 (33.5)
2	95 (17.9)
3	145 (27.3)
4	45 (8.5)
**Place of residence**	
At home	275 (51.7)
Hospital	75 (14.1)
Hospice	158 (29.7)
Nursing home	10 (1.9)
Other^e^	4 (0.8)
**Symptom score ≥ 4**	
Pain	195 (36.6)
Sleeping problems	209 (39.3)
Dry mouth	241 (45.3)
Dysphagia	111 (20.9)
Lack of appetite	274 (51.5)
Constipation	265 (49.8)
Nausea	64 (12.0)
Shortness of breath	99 (18.6)
Fatigue	344 (64.7)
Anxiety	131 (24.6)
Depressed mood	161 (30.3)

^a^Cancer site exceeds 100% because patients had more than one primary cancer site.

^b^Other primary cancer sites: Bonemarrow, M. Kahler, multiple myeloma, bone, gallbladder, cholangiocarcinoma, small intestine, anal cancer, stomach cancer, basal skin carcinoma, squamous cell carcinoma of the skin, leiomyosarcoma, sarcoma, GIST, cervical cancer, vaginal cancer, mesothelioma.

^c^Chemotherapy and/or hormone therapy and/or immunotherapy and/or other (intravenous immunoglobulin therapy, angiogenesis inhibitors, BRAF targeted therapy, unspecified targeted therapy).

^d^Patient-reported functional status^22^: 0 = normal with no limitations; 1 = not my normal self, but able to be up and about with fairly normal activities; 2 = not feeling up to most things, but in bed or chair less than half of the day; 3 = able to do little activity and spend most of the day in bed or chair; 4 = pretty much bedridden, rarely out of bed.

^e^Living facility for mentally disabled people, rehabilitation center, care home, care hotel.

### Symptom burden

Fatigue was experienced by 64.7% and 51.5% experienced lack of appetite as a clinically relevant symptom (NRS ≥ 4; see Table 1). Clinically relevant constipation was reported by 49.8% of patients, dry mouth by 45.3%, sleeping problems by 39.3% and pain by 36.6%. Clinically relevant depressed mood was reported by 30.3% of patients, anxiety by 24.6%, dysphagia by 20.9%, shortness of breath by 18.6% and nausea by 12.0%.

### Bayesian network model

Of the 9 tested automated BN algorithms, Tabu search algorithm^27^ had the highest AIC score. An overview of AIC scores for all tested algorithms is provided in Supplementary Fig. 1. When trained on the full data set, the Tabu search algorithm constructed the BN structure shown in Fig. 1. Each node represents 1 of the 11 USD-listed symptoms. In the identified BN structure, fatigue was most frequently directly associated with other USD-listed symptoms. Experiencing shortness of breath, dry mouth, anxiety, nausea, pain, sleeping problems and lack of appetite was conditionally dependent on whether a patient experienced fatigue.

The model predicted a conditional probability of > 75% of experiencing a specific simultaneous symptom based on the absence or presence of other USD-listed symptoms for the following combinations (Table 2): Patients who experienced fatigue and dysphagia had a 80.0% probability of experiencing a lack of appetite and also experiencing dysphagia and no fatigue also gave a probability of 81.0% of a lack of appetite. Experiencing dysphagia and a lack of appetite gave a 78.7% probability of experiencing constipation. Experiencing anxiety and a dry mouth gave a probability of 79.8% of a depressed mood, and also experiencing anxiety and not having a dry mouth gave a probability of 76.4% of a depressed mood.

**Table 2:** Conditional probability of the presence per simultaneous symptom based on the presence ( +) and absence (−) of main symptom(s), as estimated by the Bayesian network model.

Main symptom^a^	Main symptom^a^	Predicted simultaneous symptom	Conditional probability of experiencing simultaneous symptom (%)
Fatigue +	Sleeping problems +	Pain	54.4
Fatigue +	Sleeping problems −		37.6
Fatigue −	Sleeping problems +		40.0
Fatigue −	Sleeping problems −		13.8
Fatigue +	Anxiety +	Sleeping problems	63.5
Fatigue +	Anxiety −		41.4
Fatigue −	Anxiety +		56.3
Fatigue −	Anxiety −		18.3
Fatigue +	Sleeping problems +	Dry mouth	62.7
Fatigue +	Sleeping problems −		47.8
Fatigue –	Sleeping problems +		45.0
Fatigue −	Sleeping problems −		22.8
Dry mouth +	Nausea +	Dysphagia	54.2
Dry mouth +	Nausea −		33.0
Dry mouth −	Nausea +		31.3
Dry mouth −	Nausea −		5.8
Fatigue +	Dysphagia +	Lack of appetite	80.0
Fatigue +	Dysphagia −		56.4
Fatigue −	Dysphagia +		81.0
Fatigue −	Dysphagia −		24.4
Dysphagia +	Lack of appetite +	Constipation	78.7
Dysphagia +	Lack of appetite −		50.0
Dysphagia −	Lack of appetite +		56.8
Dysphagia −	Lack of appetite −		33.9
Fatigue +	Depressed mood +	Nausea	30.1
Fatigue +	Depressed mood −		9.5
Fatigue −	Depressed mood +		4.0
Fatigue −	Depressed mood −		1.3
Fatigue +	Dry mouth +	Shortness of breath	36.1
Fatigue +	Dry mouth −		16.0
Fatigue −	Dry mouth +		5.9
Fatigue −	Dry mouth −		2.2
–	–	Fatigue	64.7
Fatigue +		Anxiety	33.1
Fatigue −			8.6
Anxiety +	Dry mouth +	Depressed mood	78.9
Anxiety +	Dry mouth −		76.4
Anxiety −	Dry mouth +		22.9
Anxiety −	Dry mouth −		8.9

^a^ + : Symptom score on symptom assessment scale ≥  4.

^a^−: Symptom score on symptom assessment scale < 4.

### Predictive performance and calibration of the Bayesian network

**Figure 2:**
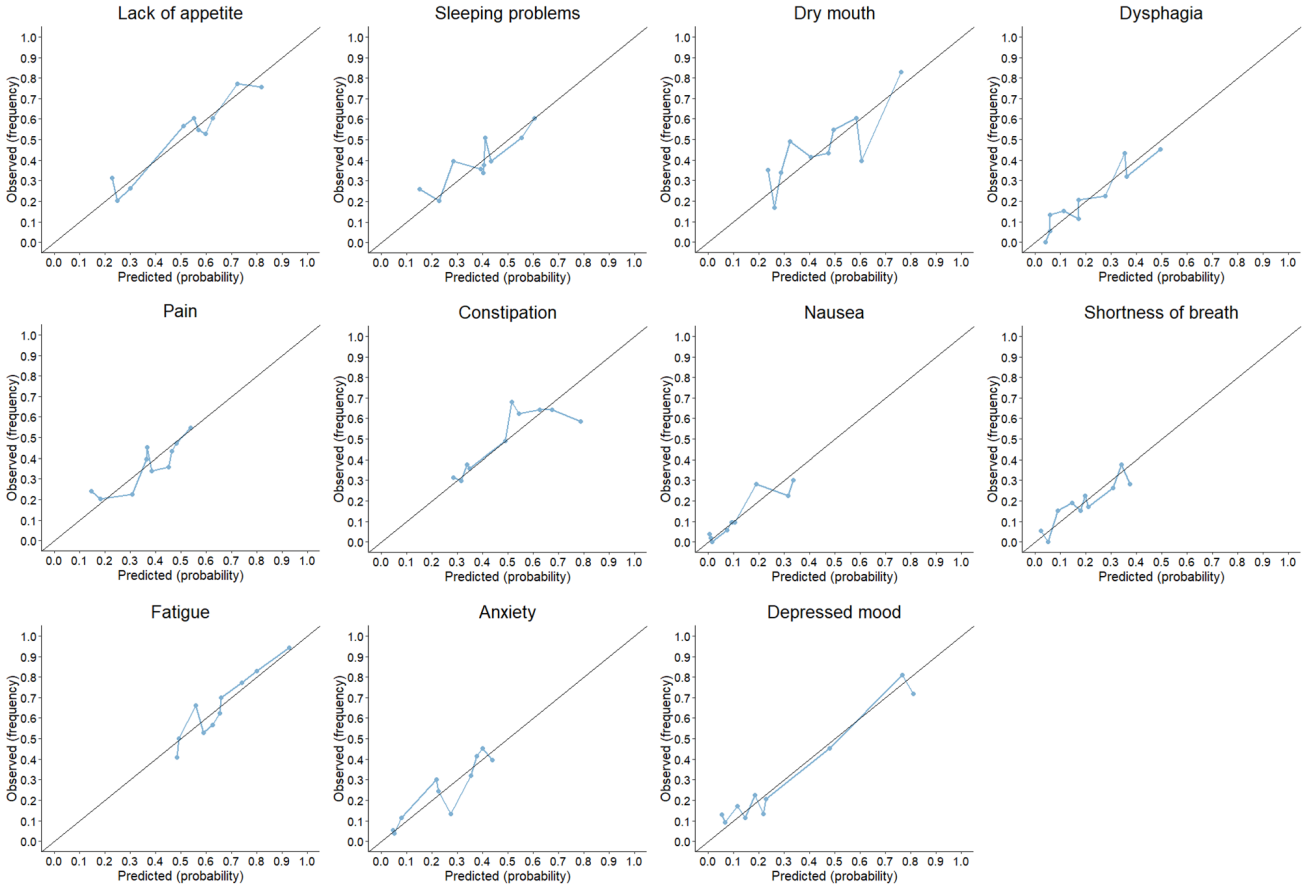
Calibration plots per predicted simultaneous symptom. The mean observed frequencies are plotted against the mean predicted probabilites per decile. Each decile represents a group of patients with a similar conditional probabilty predicted by the model. The black line corresponds to a model with ideal calibration, that is, a model that perfectly predicts the conditional probabilities.

The mean AUC-ROC per predicted symptom (see Supplementary Table 1 for AUC-ROCs per cross-validation fold) varied between 0.60 for pain and sleeping problems and 0.78 for depressed mood (Table 3). AUCs were satisfactory (≥ 0.65) for 8 out of 11 symptoms. The calibration plots show the mean predicted probabilites plotted against the mean observed frequentcies per decile (Fig. 2). The model accurately predicted 101 of a total of 110 conditional probabilities. For 7/11 symptoms, all predicted conditional probabilities were accurate at calibration. A difference of > 10% between predicted probabilities and observed frequencies was observed for sleeping problems (2 deciles), dry mouth (3 deciles), constipation (2 deciles) fatigue (1 decile) and anxiety (1 decile).

**Table3:** Predictive performance of the Bayesian network model per symptom, as measured by the mean area under the receiver operating curve calculated using fourfold cross-validation.

Predicted simultaneous symptom	AUC-ROC^a^	SD^b^
Pain	0.60	0.07
Sleeping problems	0.60	0.08
Dry mouth	0.66	0.10
Dysphagia	0.70	0.09
Lack of appetite	0.70	0.05
Constipation	0.65	0.03
Nausea	0.75	0.04
Shortness of breath	0.65	0.12
Fatigue	0.64	0.10
Anxiety	0.70	0.08
Depressed mood	0.78	0.01

*AUC-ROC* Area under the receiver operating curve, *SD* standard deviation.

^a^Mean AUC-ROC of fourfold cross-validation.

^b^Mean SD of fourfold cross-validation.

### Symptom prediction flow-chart

**Figure 3:**
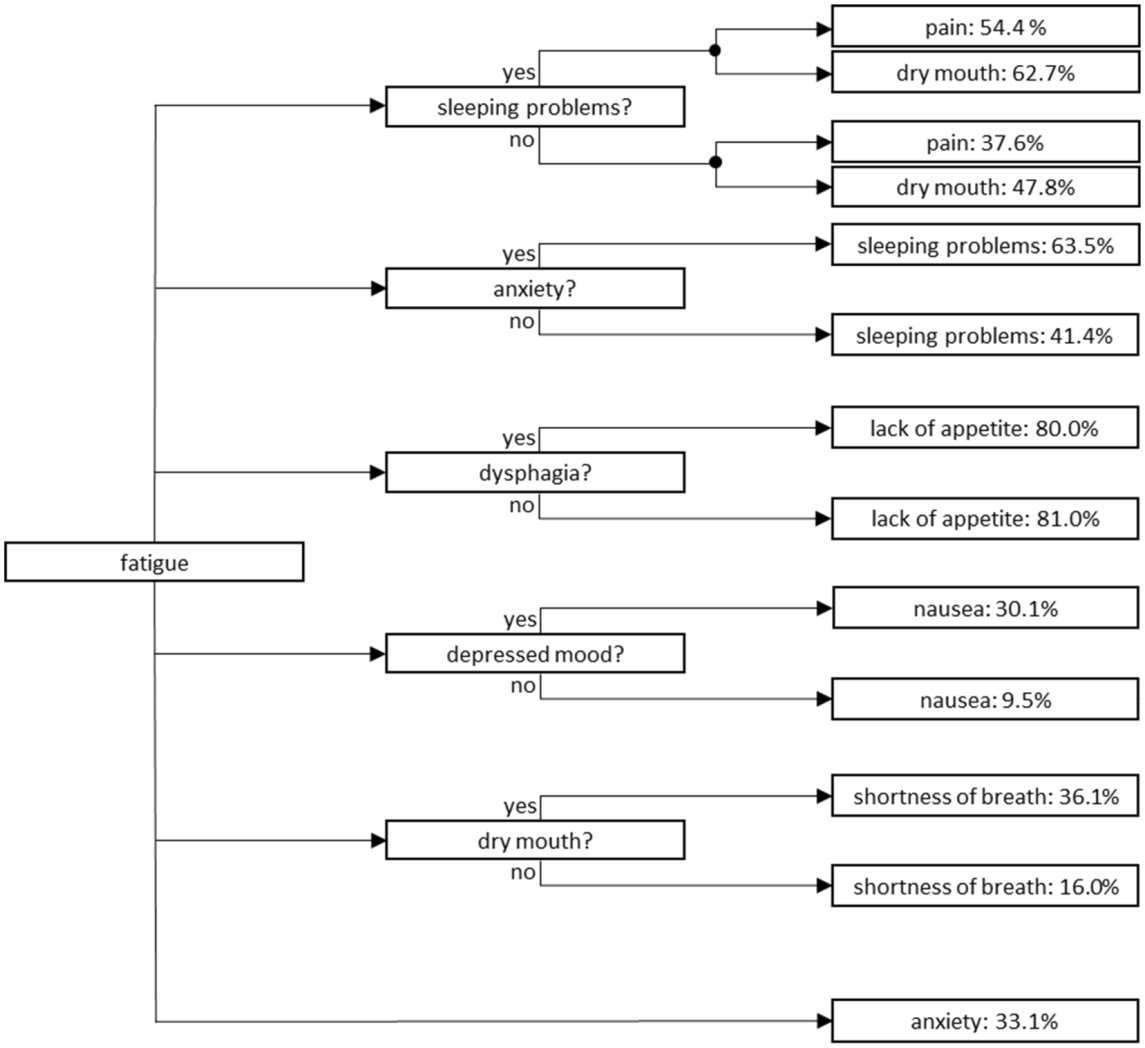
Preliminary symptom prediction system that uses the presence of fatigue as system input. Fatigue is the second most frequently volunteered symptom by patients with advanced cancer^5^, and in our Bayesian network this symptom was identified as the symptom that most frequently was directly associated with other USD-listed symptoms (7 other symptoms). When using fatigue as system input, the conditional probabilities (%) of the patient also experiencing pain, dry mouth, sleeping problems, lack of appetite, nausea, shortness of breath and anxiety can be presented. This may help clinicians to prioritize which symptoms to assess.

We propose a flow-chart (Fig. 3) based on the BN structure (Fig. 1) and conditional probabilities predicted by the BN (Table 2) to illustrate the potential of BN development for a clinical symptom prediction system. Fatigue serves as the system’s starting point because it was the symptoms that was most frequently directly associated with other symptoms and moreover, is the second most frequently volunteered symptom by patients with advanced cancer in available research^5^.

## Discussion

### Main findings

We evaluated the statistical feasibility of developing a Bayesian network (BN) for predicting the presence of simultaneous symptoms, based on the presence or absence of a patient’s other symptoms. By presenting the probability of experiencing specific simultaneous symptoms, we aim to help clinicians prioritize which symptoms to assess during consultations. The developed BN model had satisfactory performance in predicting the presence or absence of 8 out of 11 USD-listed symptoms, indicated by an AUC-ROC of ≥ 0.65. Model calibration showed that 101 out of a total of 110 conditional probabilities predicted by the BN model were accurate. Fatigue was most frequently directly associated with other USD-listed symptoms and seems most eligble to serve as the starting symptom in a future symptom prediction system.

### Identified direct associations between symptoms

All direct associations between symptoms, represented by the edges (arrows) in the BN structure, were also identified in at least one previous symptom cluster study among patients with advanced cancer. Previously identified were associations between fatigue and pain^28,29^, sleeping problems and pain^30^, fatigue and sleeping problems^30,31^, anxiety and sleeping problems^32^, dysphagia and dry mouth^33^, fatigue and anxiety^34^, fatigue and a lack of appetite^32,35,36^, dysphagia and lack of appetite^33^, dysphagia and constipation^33^, a lack of appetite and constipation^33^, fatigue and nausea^35^ dry mouth and shortness of breath^37^, fatigue and shortness of breath^31,38,39^, fatigue and anxiety^36,40^,sleeping problems and anxiety^32,37^, sleeping problems and dry mouth^41^, fatigue and dry mouth^37^, depressed mood and nausea^42^, dry mouth and depressed mood^30^, nausea and dysphagia^33^ and anxiety and depressed mood^32,35–37,39,43^.

In line with previous studies, fatigue was most frequently directly associated with other ESAS symptoms, seven in total. Fatigue is the most prevalent symptom in patients with advanced cancer, both in our study and in others^44^. Also similar to our findings, others have found that contributory factors of cancer-related fatigue include pain, sleep disturbances, emotional disturbances, depression, anxiety and weight loss^45^. Vice versa, fatigue may be a contributing factor in how severe patients experience the associated seven other ESAS symptoms. Fatigue has been found to be one of the most distressing symptoms in patients with cancer and often is accompanied by mental fatigue, including emotional liability^45,46^. These psychological factors may affect how well patients are able to cope with their other symptoms. Our ultimate aim is to use the results of a BN model for developing a clinical symptom prediction system that present clinicians with the probability that their patient experiences specific simultaneous symptoms, based on the main symptom(s) a patient volunteers during regular history taking. For this purpose, the BN model should be validated on an external dataset. External validation is a crucial step in verifying a model’s generalizibility^15^. In the external dataset, data should preferably be available on the actual volunteered main symptom(s) by patients during regular history taking. This information was absent in the current study, which likely influenced the identified assocations between symptoms. In addition to external validation, future research should aim to gain more certainty about the directions of the edges in the BN structure. This is important because the direction of the edges in the identified BN structure remains uncertain, due to the fact that automated learning was used to develop the BN. This was the only option because there was no consistent available evidence about associations between symptoms to facilitate hybrid causal learning, which means a BN model is developed based on a combination of initial expert knowledge and automated learning^47^. Automated learning may result in edges that point in a certain direction for other reasons than that it best fits the data, for example, to simplify the constructed network. When developing a final BN model to support a symptom prediction system, the probability of the edges’ directions between symptoms could be further assessed by the methods used by McNally et al., who used BN development for gaining insight in associations between the symptoms of Post Traumatic Stress Disorder^48^. They determined the direction of each edge between symptoms in 10.000 bootstrapped networks. If a direction of an edge was present in at least 51% of networks, this was considered the edge’s direction.

### Predictive preformance and model calibration

The developed BN model had an AUC-ROC of ≥ 0.65 for 8 out of 11 USD-listed symptoms. For the purpose of advising clinicians which symptoms to assess, we considered an AUC-ROC of ≥ 0.65 a satisfactory predictive performance because it is considerably better than chance (AUC-ROC = 0.5)^26^. Moreover, the consequences of symptom prediction being wrong are small, in comparison to, for example, diagnostic tests in which one generally strives for AUC scores > 0.95 because the consequences of being wrong are severe. For the same reason, we considered a difference of ≤ 10% between predicted an observed probability in model calibration acceptable.

### Potential of using Bayesian networks for symptom prediction

Using BNs to develop a symptom prediction system is a promising approach because additional variables can easily be added as the model’s variables in future research^15^, for example the patient’s age, gender, primary cancer site, functional status and disease-modifing treatment during the previous 3 months. It is likely that conditional dependency between these variables and symptoms will be identified, as others have previously identified variables such as cancer site, age and gender as predictors for differences in symptom cluster composition^8^. Inclusion of these variables may enable symptom prediction to be further individualized. In addition, problems in other than the physical and psychological dimension of palliative care could be used as variables in the BN. The Utrecht Symptom Diary (USD), a Dutch adaptation of the ESAS, was used for data analysis, which measures 11 symptoms, whereas advanced cancer patients usually experience a wider array of physical symptoms and psychological, social and existential problems^49^. Multidimensional symptoms and problems could serve as variables for future BN development to gain further insight into the complex associations between physical symptoms and non-physical problems.

The purpose of the proposed symptom prediction system is to help clinician prioritize which symptoms to assess. Since the need for prioritization will largely depend on the amount of time a clinician has available for symptom assessment, we propose that in the future system a clinician can indicate whether they have sufficient or limited time. In case of limited time, the clinician will likely only want to asses those simultaneous symptoms that are highly likely present. This could be achieved by establishing two different probability thresholds within the system, above which clinicians are advised to assess a specific simultaneous symptom. Probability thresholds can be extracted from the model’s AUC-ROC, with a specific sensitivity and specificity per threshold. If time is limited, we suggest to adhere to a threshold with relatively high specificity (low probability of false positives) and in case of sufficient time, to adhere to a threshold with high sensitivity (low probability of false negatives). When evaluating the effect of using the proposed symptom prediction system in the future, it should be noted that the ultimate problem of missing out on important symptoms is the fact that the single missed symptoms are not targeted in regular history taking and that the complex interaction of multiple symptoms is not taken into account when choosing a treatment strategy to try to relieve the patient’s total symptom burden. We therefore suggest the following positive clinical outcome of using a symptom prediction system: a significant clinically relevant relief of overall symptom burden in patients of clinicians that used the symptom prediction system compared to patients of clinicians who identified symptoms through regular history taking.

### Limitations and strenghts

In this study, a symptom assessment scale was used for data collection. To illustrate the potential of BN development we labelled symptoms as ‘main symptoms’ and ‘simultaneous symptoms’. However, we want to underline that no data were available on the actual volunteered main symptom(s) of patients. This has likely affected the identified associations between symptoms. Therefore, the current results cannot be used to present clinicians with the probability that a patient experiences specific simultaneous symptoms, based on the main volunteered symptom(s) during regular history taking.

USD scores were dichotomized into clinically relevant (≥ 4) and not relevant (< 4) since an ESAS score of ≥ 4 is generally considered as the cut off point for symptoms that require additional assessment^21,24^. Moreover, BN development is easier to illustrate using dichotomized variables, as dichotomization minimizes the number of combinations and opportunities. This dichotomization determined whether a symptom was considered present (score ≥ 4) or absent (score < 4). In future research, instead of dichotomizing the symptoms, the ESAS scores could also be categorized into severity categories (mild, moderate, severe)^50^. This way, it can be evaluated to what extent BN structure and conditional probabilites differ when ESAS scores are dichotomized or categorized. It may be argued that imputation of missing symptom scores influenced the BN structure and conditional probabilities. However, we consider this effect neglectable because only 26 symptom scores of overall 5852 symptom scores were missing in the data set. A strength of this study is that, to our knowledge, we are first to present the idea of a symptom prediction system that quantifies the probability of a patient experiencing a simultaneous symptom based on their volunteered symptoms, which we believe could be a valuable addition to the solutions already offered for improving symptom assessment in patients with advanced cancer.

## Conclusion

It is feasible to use BN development to support clinicians in prioritizing symptom assessment. The developed model was able to predict the probability that patients experience specific simultaneous symptoms based on the presence or absence of their other symptoms, given the model’s satisfactory predictive performance for 8 out of 11 symptoms. Moreover, the conditional probabilities that the BN model predicted were generally accurate. BN development could support the implementation of a symptom prediction system to help clinicians prioritize the asssesment of simultaneous symptoms in patients with advanced cancer. Fatigue seems most eligible to serves as a starting symptom of such a system. To develop a symptom prediction system, future research is needed to validate the identified model using an external dataset.

## Supplementary Information

Supplementary Information.

## Data Availability

The dataset and code used in this study is available from the corresponding author on reasonable request.
