# Supplementary material for: The feasibility of a Bayesian network model to assess the probability of simultaneous symptoms in patients with advanced cancer
Source: Sci Rep. 2022 Dec 24;12:22295. doi: 10.1038/s41598-022-26342-4 (PMC9789983; doi:10.1038/s41598-022-26342-4)
Supplement: Supplementary file 1 — Supplementary Information. [file 41598_2022_26342_MOESM1_ESM.pdf]

Supplementary figure 1. Overview of mean Akaike Information Criterion (AIC) score and standard deviation for the 9 Bayesian network algorithms, as evaluated by fourfold cross validation

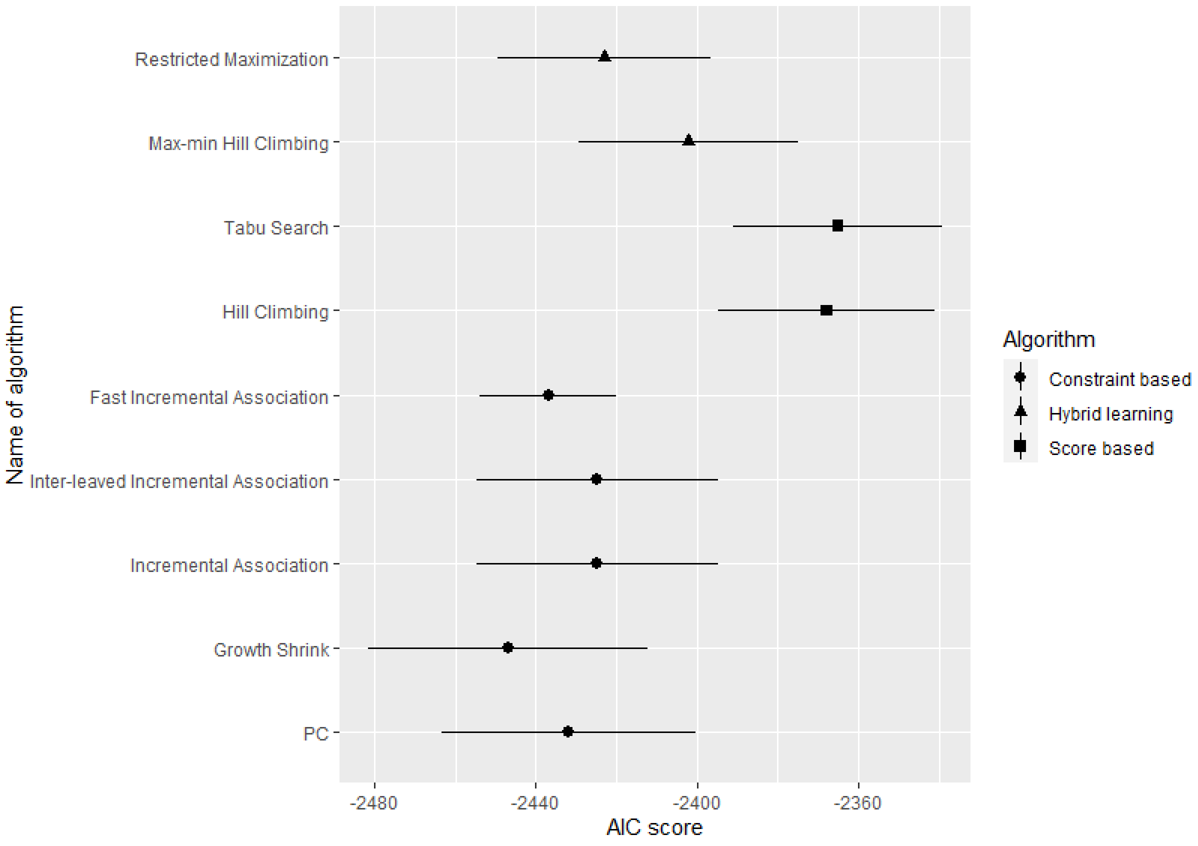

Supplementary table 1. Area Under the Curve per cross validation

	Pain	Sleeping problems	Dry mouth	Dysphagia	Lack of appetite	Constipation	Nausea	Shortness of breath	Fatigue	Anxiety	Depressed mood
AUC fold 1	0.62631184	0.68692022	0.6743373	0.80111111	0.65309815	0.64311677	0.7884154	0.5000000	0.6235294	0.69954545	0.795201485
AUC fold 2	0.67492711	0.60795181	0.5917006	0.72457951	0.65963719	0.63138851	0.7815171	0.6903307	0.5000000	0.59285714	0.785870158
AUC fold 3	0.50000000	0.59821429	0.7938228	0.57800144	0.75894475	0.61888587	0.7187875	0.7912963	0.7226620	0.78248762	0.774865591
AUC fold 4	0.60856050	0.50000000	0.5661063	0.68628293	0.71278281	0.69280822	0.7192478	0.6065688	0.7087912	0.73476454	0.782836211
Mean AUC	0.60244986	0.59827158	0.6564918	0.69749375	0.69611572	0.64654984	0.7519919	0.6470490	0.6387457	0.70241369	0.784693361
SD of AUC	0.07383689	0.07661504	0.1025438	0.09286961	0.04968727	0.03238716	0.0381799	0.1237506	0.1023560	0.08055904	0.008403166
AUC= Area Under the Curve; SD= Standard deviation											
